# Special Issue “Neuroinflammation and Neurodegeneration: Molecular Mechanism and Novel Therapy”

**DOI:** 10.3390/ijms27136027

**Published:** 2026-07-05

**Authors:** Chih Hung Lo

**Affiliations:** 1 Department of Biology, Syracuse University, Syracuse, NY 13244, USA; clo101@syr.edu; 2 Interdisciplinary Neuroscience Program, Syracuse University, Syracuse, NY 13244, USA

Neurological disorders in the form of neuroinflammation and neurodegeneration represent one of the most pressing biomedical challenges of the twenty-first century, affecting more than one in three persons in the world [1]. Disorders such as Alzheimer’s disease (AD), Parkinson’s disease (PD), substance-induced neurodegeneration, multiple sclerosis, and other autoimmune and inflammation-driven neurological disorders affect millions of individuals worldwide and impose a substantial socioeconomic burden [2]. The intricate interplay between protein aggregation, glial activation, immune signaling, metabolic impairments, vascular or blood–brain barrier (BBB) dysfunction, and systemic inflammatory processes has transformed our understanding of neurological disease pathogenesis as a multifactorial process [3,4]. Despite their diverse clinical manifestations and etiologies, a growing body of evidence suggests that the crosstalk between metabolic dysfunction [5,6] and chronic neuroinflammation [7,8] is a common pathogenic denominator that drives neuronal dysfunction, synaptic loss, and progressive neurodegeneration, giving rise to the need to elucidate and target the metabolic–inflammatory axis in neurological disorders [9,10,11].

The metabolic–inflammatory axis describes the bidirectional interplay between metabolic dysfunction and immune activation or inflammation that drives both peripheral and central nervous system (CNS) pathology [12]. Rather than representing independent processes, metabolic disturbances, including obesity, insulin resistance, dyslipidemia, and altered glucose homeostasis, promote chronic low-grade inflammation, while inflammatory signaling further disrupts cellular metabolism, creating a self-perpetuating pathogenic cycle [13,14]. Within the brain, metabolic stress impairs lysosomal function, mitochondrial activity, and proteostasis, leading to the accumulation of pathogenic intrinsically disordered protein (IDP) aggregates, activation of glial cells, blood–brain barrier (BBB) dysfunction, and impaired glymphatic clearance [15,16,17]. Outside the CNS, adipose tissue functions as an active endocrine and immune organ that releases adipokines, cytokines, chemokines, lipid mediators, extracellular vesicles, and metabolic substrates into the systemic circulation [18,19]. These circulating factors communicate with the brain by altering endothelial integrity, crossing or disrupting the BBB, and modulating neuroimmune and neuroinflammatory responses [20,21,22]. Additional peripheral contributors, including the liver, skeletal muscle, pancreas, and gut microbiota, further shape systemic metabolism and inflammatory profiles through hormones, metabolites, and microbial products. These factors influence brain function via various body–brain communication pathways such as the gut–brain, adipose–brain, and liver–brain axes [23,24]. Together, the metabolic–inflammatory axis provides a unifying framework in which systemic metabolic health, peripheral immune responses, and intrinsic brain mechanisms interact dynamically to determine susceptibility to neuroinflammation and neurodegenerative disease progression, highlighting multiple opportunities for therapeutic intervention.

This Special Issue, Neuroinflammation and Neurodegeneration: Molecular Mechanism and Novel Therapy, brings together seven contributions that collectively exemplify the multifaceted nature of neuroinflammation across neurological disorders. These studies span fundamental molecular mechanisms, disease-specific inflammatory pathways, biomarker discovery, and emerging therapeutic approaches. Together, they underscore a central theme: neurodegeneration is rarely a neuron-autonomous process. Rather, it emerges from dynamic interactions among neurons, glial cells, vascular elements, metabolic alterations, peripheral immune signals, and systemic factors that shape the neuroinflammatory landscape.

A central theme emerging from the seven contributions in this Special Issue is that neurodegeneration is driven by complex interactions among protein homeostasis, immune regulation, and systemic inflammation rather than by isolated pathological processes. Several articles illustrate how elevated tau expression and aggregation disrupt cytoskeletal networks and intracellular transport within the tau-microtubule-end-binding protein axis [25] and under an autoimmune-associated anti-IgLON5 disease condition [26], illustrating the convergence of protein aggregation, cytoskeletal dysfunction, and immune-mediated pathology. Complementing these mechanistic insights, multiple studies emphasize neuroimmune communication as a key determinant of disease progression. Therapeutic strategies aimed at modulating microglial function through CX3CR1-engineered regulatory T cells [27], promotion of neuroprotective microglial phenotypes with abscisic acid [28], or inhibition of P2X7 receptor signaling [29] demonstrate the growing shift from broad immunosuppression toward precision immunomodulation that restores immune homeostasis while preserving protective immune functions. Extending beyond the CNS, other contributions underscore the importance of systemic factors in neurological diseases. The review on the gut–brain axis in PD indicates how intestinal dysbiosis and peripheral inflammation influence neurodegeneration [30], whereas studies of inflammatory biomarkers following aneurysmal subarachnoid hemorrhage reveal the dynamic and temporally evolving nature of neuroinflammatory responses after acute brain injury [31]. Altogether, these studies demonstrate an emerging paradigm in which neurodegenerative diseases are viewed as disorders involving interconnected cellular, immune, vascular, and peripheral systems, thereby providing new mechanistic insights and identifying diverse therapeutic opportunities for targeting neuroinflammation across a broad spectrum of neurological disorders.

**Microtubule destabilization as an initiator of organelle transport defect and proteostasis failure**

One emerging theme discussed in this Special Issue is that disruption of the neuronal cytoskeleton represents an early pathogenic event that extends well beyond structural instability. The review on the tau-microtubule-end-binding protein axis illustrates how pathological tau impairs microtubule dynamics, disrupting intracellular trafficking that is essential for neuronal homeostasis [25]. As microtubules serve as the primary transport network for lysosomes, mitochondria, endosomes, autophagosomes, and synaptic vesicles, their dysfunction compromises organelle positioning, axonal transport, protein turnover, and synaptic maintenance [32,33,34]. Emerging evidence further indicates that impaired lysosomal acidification, transport, and maturation contribute to defective autophagy and the accumulation of pathogenic proteins, including tau, β-amyloid, and α-synuclein [35,36,37,38], thereby amplifying cellular stress. Similarly, the review on anti-IgLON5-associated disease demonstrates that immune-mediated tau pathology converges with cytoskeletal disruption, reinforcing the notion that neurodegeneration arises through interconnected rather than isolated mechanisms [26,39]. Together, these studies support an evolving model in which cytoskeletal dysfunction initiates widespread defects in intracellular trafficking, organelle communication, and proteostasis [40], ultimately creating a metabolically stressed neuronal environment that predisposes the brain to chronic neuroinflammation and progressive neurodegeneration.

**Neuroinflammation and metabolic dysfunction form a self-perpetuating pathogenic cycle**

Several studies in this collection further emphasize that neuroinflammation and metabolic dysfunction are tightly interconnected rather than independent pathological processes. Chronic activation of microglia is accompanied by profound metabolic reprogramming that reinforces inflammatory signaling while impairing neuronal homeostasis [41]. The studies examining CX3CR1-transduced regulatory T cells in AD [27], the effects of abscisic acid on microglial activation [28], and P2X7 receptor signaling during chronic ethanol exposure [29] demonstrate that manipulating immune responses can restore metabolic balance and reduce cellular stress within the CNS. P2X7R acts as a sensor of extracellular adenosine triphosphate released during cellular stress, linking metabolic perturbations to inflammasome activation, BBB disruption, and cognitive decline [29]. Conversely, regulatory T cells and abscisic acid promote neuroprotective immune phenotypes that suppress excessive cytokine production while preserving tissue repair [27,28], whereas inflammatory profiling following aneurysmal subarachnoid hemorrhage reveals temporally dynamic cytokine responses that shape subsequent neurological outcomes [31]. These studies support the concept that inflammation not only results from metabolic dysfunction but also actively disrupts cellular metabolism and neuronal survival. Such reciprocal interactions define the metabolic–inflammatory axis, in which metabolic stress and immune activation continuously reinforce one another to accelerate neuroinflammation and neurodegeneration.

**The metabolic–inflammatory axis extends beyond the brain through body–brain communication**

An equally important concept emerging from this Special Issue is that neurodegeneration cannot be understood solely as a CNS disorder but instead reflects continuous bidirectional communication between peripheral organs and the brain. The review on fecal microbiota transplantation in PD exemplifies this body–brain interaction by demonstrating how alterations in gut microbial composition reshape host metabolism, intestinal barrier integrity, immune activation, and circulating metabolites that subsequently influence neuroinflammation and α-synuclein pathology [30]. Likewise, the therapeutic efficacy of adoptively transferred CX3CR1-engineered regulatory T cells illustrates how peripheral immune cells can migrate into the brain and re-establish neuroimmune homeostasis [27]. These findings fit within the broader framework of the metabolic–inflammatory axis, whereby adipose tissue, liver, skeletal muscle, and the gut function as metabolically active endocrine and immune organs that release cytokines, chemokines, adipokines, lipid mediators, metabolites, and extracellular vesicles into the circulation. These systemic signals influence BBB integrity, glial activation, metabolic activities, and neuronal function, while pathological processes within the brain reciprocally alter peripheral immune and metabolic states [42,43]. Together, these studies strengthen an integrated systems biology perspective in which neurodegenerative diseases arise through dynamic interactions among intracellular proteostatic failure, neuroimmune dysregulation, and systemic metabolic inflammation [20,21,22]. This holistic framework offers new opportunities for therapeutic intervention by targeting multiple nodes of the metabolic–inflammatory axis rather than individual pathological pathways.

Collectively, the contributions in this Special Issue highlight a growing paradigm shift in neurodegeneration research: neurological disorders should no longer be viewed as the consequence of isolated protein aggregation, immune dysregulation, or metabolic impairment, but rather as diseases arising from the dynamic integration of these processes across multiple biological scales. The metabolic–inflammatory axis provides a unifying framework that links intracellular dysfunction, including cytoskeletal instability, impaired organelle trafficking, mitochondrial and lysosomal dysfunction, insulin resistance, and defective proteostasis, with chronic neuroinflammation and systemic metabolic disturbances. Importantly, this axis extends beyond the brain through continuous communication with peripheral organs, including adipose tissue, the liver, skeletal muscle, and the gut, whose metabolites, cytokines, hormones, extracellular vesicles, and immune cells shape the neuroimmune environment. Conversely, pathological events within the CNS such as IDP aggregation can propagate systemic metabolic and inflammatory alterations, establishing self-amplifying feedback loops that accelerate disease progression. Rather than representing independent pathological pathways, these interconnected mechanisms determine neuronal resilience or vulnerability throughout aging as well as heterogeneous phenotypes across populations [44,45,46]. As our understanding of the metabolic–inflammatory axis continues to evolve, future therapies will likely move beyond single-target interventions toward re-establishing cellular homeostasis by simultaneously promoting toxic protein degradation [47,48], restoring organelle function [49,50], reducing inflammation and immune imbalance [51,52,53], and correcting systemic metabolic abnormalities [54,55], thereby offering more effective strategies for attenuating neuroinflammation and neurodegeneration under various neurological disorders.

## Data Availability

No new data were created or analyzed in this study. Data sharing is not applicable to this article.
